# The Utility of SMS to Report Male Partner HIV Self-testing Outcomes Among Women Seeking Reproductive Health Services in Kenya: Cohort Study

**DOI:** 10.2196/15281

**Published:** 2020-03-25

**Authors:** Alison L Drake, Emily Begnel, Jillian Pintye, John Kinuthia, Anjuli D Wagner, Claire W Rothschild, Felix Otieno, Valarie Kemunto, Jared M Baeten, Grace John-Stewart

**Affiliations:** 1 Department of Global Health University of Washington Seattle, WA United States; 2 School of Nursing University of Washington Seattle, WA United States; 3 Research and Programs Kenyatta National Hospital Nairobi Kenya; 4 Department of Epidemiology University of Washington Seattle, WA United States; 5 Department of Medicine University of Washington Seattle, WA United States; 6 Department of Pediatrics University of Washington Seattle, WA United States

**Keywords:** SMS, HIV self-testing, survey coverage, HIV pre-exposure prophylaxis

## Abstract

**Background:**

Use of SMS for data collection is expanding, but coverage, bias, and logistical constraints are poorly described.

**Objective:**

The aim of this study is to assess the use of SMS to capture clinical outcomes that occur at home and identify potential biases in reporting compared to in-person ascertainment.

**Methods:**

In the PrEP Implementation in Young Women and Adolescents program, which integrated pre-exposure prophylaxis (PrEP) into antenatal care, postnatal care, and family planning facilities in Kisumu County, Kenya, HIV-negative women 14 years of age or older were offered oral HIV self-tests (HIVSTs) to take home to male partners. Women that brought a phone with a Safaricom SIM to the clinic were offered registration in an automated SMS system (mSurvey) to collect information on HIVST outcomes. Women were asked if they offered the test to their male partners, and asked about the test process and results. HIVST outcomes were collected via SMS (sent 2.5 weeks later), in-person (if women returned for a follow-up scheduled 1 month later), or using both methods (if women initiated PrEP, they also had scheduled follow-up visits). The SMS prompted women to reply at no charge. HIVST outcomes were compared between women with scheduled follow-up visits and those without (follow-up visits were only scheduled for women who initiated PrEP). HIVST outcomes were also compared between women reporting via SMS and in-person.

**Results:**

Among 2123 women offered HIVSTs and mSurvey registration, 486 (23.89%) accepted HIVSTs, of whom 359 (73.87%) were eligible for mSurvey. Additionally, 76/170 (44.7%) women with scheduled follow-up visits and 146/189 (77.3%) without scheduled follow-up visits registered in mSurvey. Among the 76 women with scheduled follow-ups, 62 (82%) had HIVST outcomes collected: 19 (31%) in-person, 20 (32%) by SMS, and 23 (37%) using both methods. Among the 146 women without scheduled visits, 87 (59.6%) had HIVST outcomes collected: 3 (3%) in-person, 82 (94%) by SMS, and 2 (2%) using both methods. SMS increased the collection of HIVST outcomes substantially for women with scheduled follow-up visits (1.48-fold), and captured 82 additional reports from women without scheduled follow-up visits. Among 222 women with reported HIVST outcomes, frequencies of offering partners the HIVST (85/95, 89% in-person vs 96/102, 94% SMS; *P*=.31), partners using the HIVST (83/85, 98% vs 92/96, 96%; *P*=.50), women using HIVST with partners (82/83, 99% vs 91/92, 99%; *P*=.94), and seeing partner’s HIVST results (82/83, 99% vs 89/92, 97%; *P*=.56) were similar between women reporting in-person only versus by SMS only. However, frequency of reports of experiencing harm or negative reactions from partners was more commonly reported in the SMS group (17/102, 16.7% vs 2/85, 2%; *P*=.003). Barriers to the SMS system registration included not having a Safaricom SIM or a functioning phone.

**Conclusions:**

Our results suggest that the use of SMS substantially improves completeness of outcome data, does not bias reporting of nonsensitive information, and may increase reporting of sensitive information.

## Introduction

SMS has enormous potential for public health. This technology is inexpensive and has increasing reach with expanding global cellular network coverage and phone ownership. SMS has been used to provide reminders [1,2], return lab results [3], provide education and improve knowledge [4], and promote healthy behaviors [5,6]. SMS has also been used to remotely collect survey data on health outcomes and behaviors, a strategy that may reduce the travel time to and cost of follow-up visits, and allows participation at convenient times [7].

Studies in low- and middle-income countries comparing SMS to other data collection approaches or offering choices in approaches are limited [8-10]. Some individuals may be able to overcome barriers to in-person visits, or may find that using both SMS and in-person approaches to survey assessment is acceptable and feasible. However, SMS may also be useful for individuals who might otherwise decline participation or become lost to follow-up. Self-administered surveys may reduce social desirability bias [11-13], although differential outcome ascertainment may bias results by using multiple approaches to data collection. Using a combination of strategies to capture health outcomes may improve participation and generalizability, but it is important to measure outcomes using different strategies in the same context and setting to determine whether results are biased based on the strategy used. We measured the utility of incorporating SMS as an alternative, complementary strategy to in-person assessment of male partner HIV self-test (HIVST) outcomes by women, and assessed bias in reporting results using either method.

## Methods

From November 20, 2017, to June 15, 2018, 3425 women seeking antenatal care (ANC), postpartum care (PNC), or family planning (FP) services at 8 facilities in Kisumu County, Kenya were asked to take an HIVST home to their male partners as part of a pre-exposure prophylaxis (PrEP) implementation program. Women were offered PrEP, and those that accepted had a PrEP follow-up visit scheduled 1 month later where HIVST outcomes were ascertained in-person. Women were classified as having scheduled follow-up visits if they initiated PrEP. Women who declined PrEP but still took an HIVST had outcomes ascertained in-person if they returned for maternal and child health or family planning services [14].

A subset of women who agreed to take an HIVST were asked to register in an automated SMS communication system (mSurvey; Nairobi, Kenya) to assess HIVST outcomes. Registration was offered in all program sites over time (initially only offered by 1 site, but expanded to all 8 sites by the end of program activity) for eligible women. Women were eligible to register if they had their phone at the clinic and a Safaricom SIM card, and provided oral consent. Registered women selected their preferred language (English, Kiswahili, or Dholou) and were asked to save the mSurvey phone number as a contact in their phone to ensure the phone number would be recognized when the follow-up survey was sent (2.5 weeks later). Women were informed that responding to the mSurvey SMS was free, and they could opt-out at any time. HIVST outcomes were assessed through sequential SMS inquiries sent by mSurvey for women to respond to using numerical responses representing survey answers. Women who initiated registration or follow-up surveys were given 72 hours to complete surveys before they were timed-out of the system and unable to complete the survey.

Mode of HIVST outcome ascertainment (SMS vs in-person) was compared between women with and without scheduled follow-up visits. HIVST outcomes were compared between SMS and in-person responders. The primary analysis was restricted to women offered HIVSTs at facilities when mSurvey registration was available. Women who reported having a partner with HIV at enrollment were excluded from the analysis. Continuous and categorical variables were compared using the Wilcoxon rank-sum and chi-square tests, respectively. Statistical analyses were performed using Stata v14 (StataCorp LLC; College Station, TX).

This study was reviewed and approved by the University of Washington Institutional Review Board and the Kenyatta National Hospital Ethics & Research Committee.

## Results

**Figure 1:**
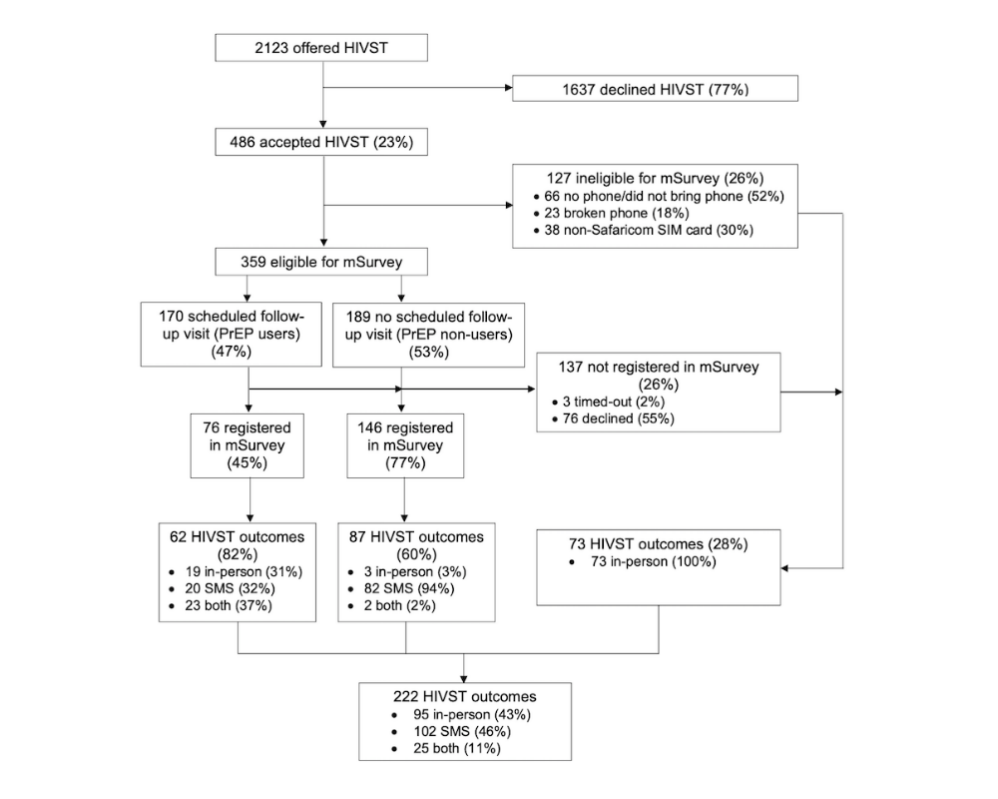
Flow diagram of women accepting HIV self-tests for male partners. HIVST: HIV self-test; PrEP: pre-exposure prophylaxis.

In total, 486 women accepted HIVSTs; 359 were eligible for registration in the mSurvey SMS system, and 222 successfully registered (Figure 1). Some women were ineligible due to the logistical barriers of not having a phone, not having a working phone, or not having a Safaricom SIM card. Nearly half (98/222, 43.6%) of the women who successfully registered for mSurvey timed-out before completing the follow-up SMS survey.

Overall, among 222 women enrolled in mSurvey: 116 (52.3%) were from ANC, 80 (36.0%) were from PNC, and 26 (11.7%) were from FP. The median age was 25 (IQR 22-28); 86.4% (191/222) were married and 17.1% (38/222) knew their male partners were HIV-negative. Intimate partner violence (IPV) was experienced by 4.5% (10/222) of the enrolled women within the last 6 months. HIVST outcomes were more likely to be reported through SMS only (vs in-person only), by women who were older (median 25 vs 23 years of age; *P*=.01), or if they had a partner of unknown HIV status (92/102, 90.2% vs 72/95, 76%; *P*=.01). Marital status (*P*=.84), history of IPV (*P*=.16), and risk factors for HIV (transactional sex, *P*=.52; diagnosis with or treatment for STI, *P*=0.14; forced sex, *P*=0.14; shared needles while engaging in intravenous drug use, *P*=0.14; and used post-exposure prophylaxis more than twice, *P*=0.14) were similar between women with HIVST outcomes assessed in-person and via SMS.

Of the 76 women registered in mSurvey with scheduled follow-up visits, 82% (62) had HIVST outcome data available. SMS increased outcome ascertainment 1.48-fold (relative risk; CI 1.32-1.64); an additional 32% of HIVST outcomes would have been missed without SMS. The majority of women enrolled in mSurvey but without scheduled follow-up visits (82/87, 94%) reported HIVST outcomes by SMS only. SMS responses captured 102/149 (68.5%) of all HIVST outcomes assessed by women in mSurvey.

HIVST outcomes were also assessed for 73 women who were ineligible or not registered in mSurvey. The frequency of reporting on HIVST outcomes by women with scheduled follow-up visits who were ineligible (32/46, 70%) or who did not register in mSurvey (41/94, 44%) was similar to the frequency of women who registered in mSurvey. Most HIVST outcomes were reported near the time SMS surveys were sent to women using SMS (median 0 days after SMS delivered, maximum 46 days), or near the scheduled follow-up date for women reporting in-person (median 0 days after scheduled date, IQR –2 to 5, range –29 to 141).

Table 1 shows the data collected from reports on HIVST experiences. The offer of HIVSTs to partners was similar between women reporting in-person and via SMS. There were no differences in reporting between in-person and SMS regarding whether or not partners took the test and if the women saw the results. A history of IPV was lower in women who reported HIVST outcomes by SMS vs in-person (3/102, 2.9% vs 7/95, 7%; *P*=.13). However, reports of experiencing harm or negative reactions from partners as a result of the HIVST were significantly more frequent in women with HIVST outcomes assessed via SMS than in-person.

**Table 1:** Partner HIV self-testing experience reports.

		Overall, n (%)	In-person, n (%)	SMS, n (%)	Both, n (%)	*P* value^a^
**Offered partner HIVST** ^b^		N=222	N=95	N=102	N=25	.31
	No	15 (6.8)	9 (9.5)	5 (4.9)	1 (4.0)	
	Yes	205 (92.3)	85 (89.5)	96 (94.1)	24 (96.0)	
	Refused to answer/Don’t know	2 (0.5)	1 (1.1)	1 (1.0)	0 (0.0)	
**Reasons partner HIVST not offered**		N=10	N=9	—^c^	N=1	—
	Fear of partner’s reaction	1 (10.0)	0 (0.0)	—	1 (100.0)	
	Have not seen partner since HIVST received	3 (30.0)	3 (33.3)	—	0 (0.0)	
	Tried to discuss, partner reacted negatively	4 (40.0)	4 (44.4)	—	0 (0.0)	
	Other	2 (20.0)	2 (22.2)	—	0 (0.0)	
Partner used HIVST		N=205	N=85	N=96	N=24	.50
		199 (97.1)	83 (97.7)	92 (95.8)	1 (100.0)	
Tested with partner		N=199	N=83	N=92	N=24	.94
		197 (99.0)	82 (98.8)	91 (98.9)	24 (100.0)	
**Saw partner's results**		N=199	N=83	N=92	N=24	.56
	Yes, I observed it	195 (98.0)	82 (98.8)	89 (96.7)	24 (100.0)	
	No, I was told the results	3 (1.5)	1 (1.2)	2 (2.2)	0 (0.0)	
	No, I don’t know his result	1 (0.5)	0 (0.0)	1 (1.1)	0 (0.0)	
**Partner HIV results**		N=198	N=83	N=91	N=24	.52
	HIV-negative	190 (96.0)	80 (96.4)	87 (95.6)	23 (95.8)	
	HIV-positive	4 (2.0)	2 (2.4)	1 (1.1)	1 (4.2)	
	Refused to answer	2 (1.0)	1 (1.2)	1 (1.1)	0 (0.0)	
	Missing	2 (1.0)	0 (0.0)	2 (2.2)	0 (0.0)	
**Experienced harm as a result of the HIVST**		N=222	N=95	N=102	N=25	<.01
	No	190 (85.6)	85 (89.5)	80 (78.4)	25 (100.0)	
	Yes	19 (8.6)	2 (2.1)	17 (16.7)	0 (0.0)	
	Refused to answer	1 (0.4)	0 (0.0)	1 (1.0)	0 (0.0)	
	Missing	12 (5.4)	8 (8.4)	4 (3.9)	0 (0.0)	

^a^Comparing in-person and SMS groups.

^b^HIVST: HIV self-test.

^c^SMS reporting results not evaluated, and *P* value not determined.

## Discussion

We found that nearly half of women seeking reproductive health services were willing and able to use SMS to respond to surveys on sensitive HIVST outcomes for their male partners. The SMS-based survey substantially increased the proportion of women that reported HIVST outcomes among women with scheduled follow-up visits (1.48-fold increase), and was a successful strategy to capture HIVST outcomes for those without scheduled follow-up visits. We did not detect differences in the proportions of women who reported that partners used HIVSTs or reported their partner’s HIV status; however, we did note a significant difference in the frequency of reported social harm related to HIVSTs. This suggests that the mode of outcome ascertainment did not bias responses for nonsensitive questions and may have improved the reporting of sensitive information, such as reports of social harm related to HIVSTs.

Our analysis was subject to some limitations, but also provided insight on logistical barriers to SMS registration. Restricting registration to the primary mobile carrier and requiring phones to be present led to exclusions of 21.3% (104/486) of women accepting HIVSTs. We were unable to disaggregate lack of phone ownership from phones not being brought to the clinic. Women in our study may also have had intermittent access to a mobile phone due to shared phones with male partners or other community members, which have previously been reported as potential barriers to including women in SMS surveys [15]. System time-out at registration was uncommon, but 43.6% (96/222) timed-out at follow-up and did not complete the survey. These findings may indicate poorer network coverage and power supply problems outside the clinic, or that participants became disinterested or uncomfortable answering the SMS surveys. Mobile phone accessibility, network coverage consistency, and difference in mobile carriers may impact generalizability and should be considered during the development stage of SMS projects [15]. Alternative strategies for survey registration, such as remote registration for individuals who share phones or do not have their phone present, may improve eligibility for SMS surveys. Finally, this study may have lacked power to compare some HIVST outcomes between women responding via SMS and in-person, such as partners with HIV, and was not able to compare reasons for not offering HIVSTs since this information was not collected via SMS.

SMS has successfully been used to remotely collect survey data [16-20]. Studies suggest that participants are willing to respond to sensitive questions if reminders to delete messages, passwords, or personal identification numbers are used [21,22]. In East Africa, SMS response rates to surveys on sexual behaviors, pregnancy history, HIV testing, and adherence to PrEP range between 14% and 96% [16,21,23-25]. Response rates increase with financial incentives, clear instructions for responding, and in the context of research [17,21,26]. Lack of incentives coupled with misconceptions that costs would be incurred for SMS responses may have hindered response rates in our program compared to other studies [27,28].

In conclusion, SMS enhanced our ability to measure male partner HIVST outcomes. Our study demonstrates that SMS can be used to collect brief survey data on sensitive information, even in the absence of financial incentives. SMS should be considered to capture health outcomes, which may alleviate health system constraints and burdens associated with in-person visits.

## Abbreviations

ANC: antenatal care
FP: family planning
HIVST: HIV self-test
IPV: intimate partner violence
PNC: postpartum care
PrEP: pre-exposure prophylaxis.
